# hERG1 behaves as biomarker of progression to adenocarcinoma in Barrett's esophagus and can be exploited for a novel endoscopic surveillance

**DOI:** 10.18632/oncotarget.11149

**Published:** 2016-08-09

**Authors:** Elena Lastraioli, Tiziano Lottini, Jessica Iorio, Giancarlo Freschi, Marilena Fazi, Claudia Duranti, Laura Carraresi, Luca Messerini, Antonio Taddei, Maria Novella Ringressi, Marianna Salemme, Vincenzo Villanacci, Carla Vindigni, Anna Tomezzoli, Roberta La Mendola, Maria Bencivenga, Bruno Compagnoni, Mariella Chiudinelli, Luca Saragoni, Ilaria Manzi, Giovanni De Manzoni, Paolo Bechi, Luca Boni, Annarosa Arcangeli

**Affiliations:** ^1^ Department of Experimental and Clinical Medicine, University of Florence, 50134 Florence, Italy; ^2^ Department of Surgery and Translational Medicine, University of Florence, 50134 Florence, Italy; ^3^ DI.V.A.L Toscana Srl, 50019 Sesto Fiorentino, Italy; ^4^ Institute of Pathology, Spedali Civili, 25123 Brescia, Italy; ^5^ Pathology Division, Azienda Ospedaliero-Universitaria Senese, 53100 Siena, Italy; ^6^ Pathology Division, Borgo Trento Hospital, 37134 Verona, Italy; ^7^ Division of Surgery, University of Verona, 37134 Verona, Italy; ^8^ Surgery Division, Esine Hospital, ASL Vallecamonica Sebino, 25040 Esine (BS), Italy; ^9^ Pathology Division, Esine Hospital, ASL Vallecamonica Sebino, 25040 Esine (BS), Italy; ^10^ Pathology Division, Morgagni-Pierantoni Hospital, 47121 Forlì, Italy; ^11^ Gastroenterology and Endoscopy Unit, Morgagni-Pierantoni Hospital, 47121 Forlì, Italy; ^12^ Clinical Trials Coordinating Center, Azienda Ospedaliero-Universitaria Careggi/Istituto Toscano Tumori, 50134 Florence, Italy

**Keywords:** hERG1, Barrett's esophagus, adenocarcinoma progression, surveillance, optical imaging

## Abstract

Barrett's esophagus (BE) is the only well-known precursor lesion of esophageal adenocarcinoma (EA). The exact estimates of the annual progression rate from BE to EA vary from 0.07% to 3.6%. The identification of BE patients at higher risk to progress to EA is hence mandatory, although difficult to accomplish. In search of novel BE biomarkers we analyzed the efficacy of hERG1 potassium channels in predicting BE progression to EA. Once tested by immunohistochemistry (IHC) on bioptic samples, hERG1 was expressed in BE, and its expression levels increased during progression from BE to esophageal dysplasia (ED) and EA. hERG1 was also over-expressed in the metaplastic cells arising in BE lesions obtained in different BE mouse models, induced either surgically or chemically. Furthermore, transgenic mice which over express hERG1 in the whole gastrointestinal tract, developed BE lesions after an esophago-jejunal anastomosis more frequently, compared to controls. A case-control study was performed on 104 bioptic samples from newly diagnosed BE patients further followed up for at least 10 years. It emerged a statistically significant association between hERG1 expression status and risk of progression to EA. Finally, a novel fluorophore- conjugated recombinant single chain variable fragment antibody (scFv-hERG1-Alexa488) was tested on freshly collected live BE biopsies: it could recognize hERG1 positive samples, perfectly matching IHC data.

Overall, hERG1 can be considered a novel BE biomarker to be exploited for a novel endoscopic surveillance protocol, either in biopsies or through endoscopy, to identify those BE patients with higher risk to progress to EA.

## INTRODUCTION

Barrett's esophagus (BE) is a pathologic condition easily detectable at endoscopy and characterized by the replacement of the squamous epithelium of the distal esophagus by a columnar-type mucosa (i.e. intestinal metaplasia), when biopsied [1]. The real prevalence of BE in the general population varies from 1.6 to 5.6%, depending on demographic features (sex, age, ethnicity) together with geographical variations [2]. Patients with BE have an increased risk to develop an esophageal adenocarcinoma (EA) [3]. Indeed BE is the only well-known pathologic precursor of EA, a process often preceded by the occurrence of dysplasia (esophageal dysplasia, ED), with a stepwise progression from low grade to high grade dysplasia, and adenocarcinoma. The wide variation in the estimates of the annual progression rate of BE to EA (0.07%–3.6%) makes it mandatory to perform endoscopic surveillance to all BE patients. However, endoscopy is a highly invasive, low cost-effective procedure. Moreover, endoscopic detection of dysplasia is a late diagnostic option, affected by multiple biases such as sampling error and subjective evaluation. Overall, the identification of BE patients at higher risk to progress to EA is difficult [4].

For these reasons, the search of BE biomarkers is of capital importance. Several potential biomarkers have been identified, and some of them have been proposed for proper BE diagnosis (e.g. c-MYC, HER2 [5]), others for prediction of progression (EGFR [6], P53 [7]) or for prognosis (miRNA profile [8]). Since none of the above biomarker has *per se* a clear cut valence for either diagnostic or prognostic purposes, the use of molecular panels (reviewed by [9]) has been proposed. For example, a panel of three molecular markers (P53, cyclin A and aneuploidy) has been proven to ensure a precise and objective diagnosis [10]. Recently, Fassan and coworkers [11] showed that the overall expression of transcribed ultra-conserved regions (T-UCR), which is altered in BE, ED and EA, might represent a potential novel molecular panel in esophageal neoplastic pathologies.

A proper screening protocol should lead to the identification of BE and ED lesions with the highest accuracy and the minimum discomfort for the patient. Hence biomarkers would be extremely useful if associated to high resolution imaging techniques (reviewed in [12]). To this purpose, several improvements to endoscopy, which represents the gold standard for BE surveillance and screening, have been performed. Indeed Chromoendoscopy and Narrow Banding Imaging (NBI), as well as the exploitation of autofluorescence due to endogenous fluorophores, greatly improved the quality of endoscopy images and hence the accuracy of diagnosis [13].

In the last twenty years the involvement of ion channels and transporters (ICTs) in tumor development and progression has gathered much attention (reviewed in [14]). ICTs represent novel cancer biomarkers as well as therapy targets. In esophageal pathologies, the potassium channel EAG and the non-selective cation channel TRPC have been identified as negative prognostic factors in squamous esophageal cancers (reviewed in [14]). Conversely, the apical sodium-dependent bile acid transporters (SLC10A2) is highly expressed in BE and EA compared to normal esophagus whereas the overexpression of the divalent metal transporter1 (SLC11A2) is associated with metastatization in EA [14].

A peculiar role in neoplastic lesions of the upper gastrointestinal tract is exerted by hERG1 potassium channels. In particular, hERG1 is overexpressed in gastric cancers, where it contributes to increase VEGF-A secretion. hERG1 expression also identifies a subgroup of patients that might be treated with a combined therapy, using hERG1 inhibitors and anti-angiogenetic drugs [15]. In a small cohort of patients, hERG1 was found to be expressed in BE, while absent in normal esophageal mucosa and in gastro-esophageal reflux disease (GERD), with or without esophagitis [16].

Moving down this line, we designed the present study aimed at: (i) confirming hERG1 expression in a larger cohort of BE patients using an antibody which binds to a different, extracellular, epitope of the hERG1 protein; (ii) determining the expression of hERG1 during BE progression to ED and EA; (iii) testing the possibility of considering hERG1 as a progression marker in BE to be further exploited for BE surveillance screenings.

## RESULTS

### hERG1 is expressed in BE and its expression increases during esophageal tumor progression

**Figure 1:**
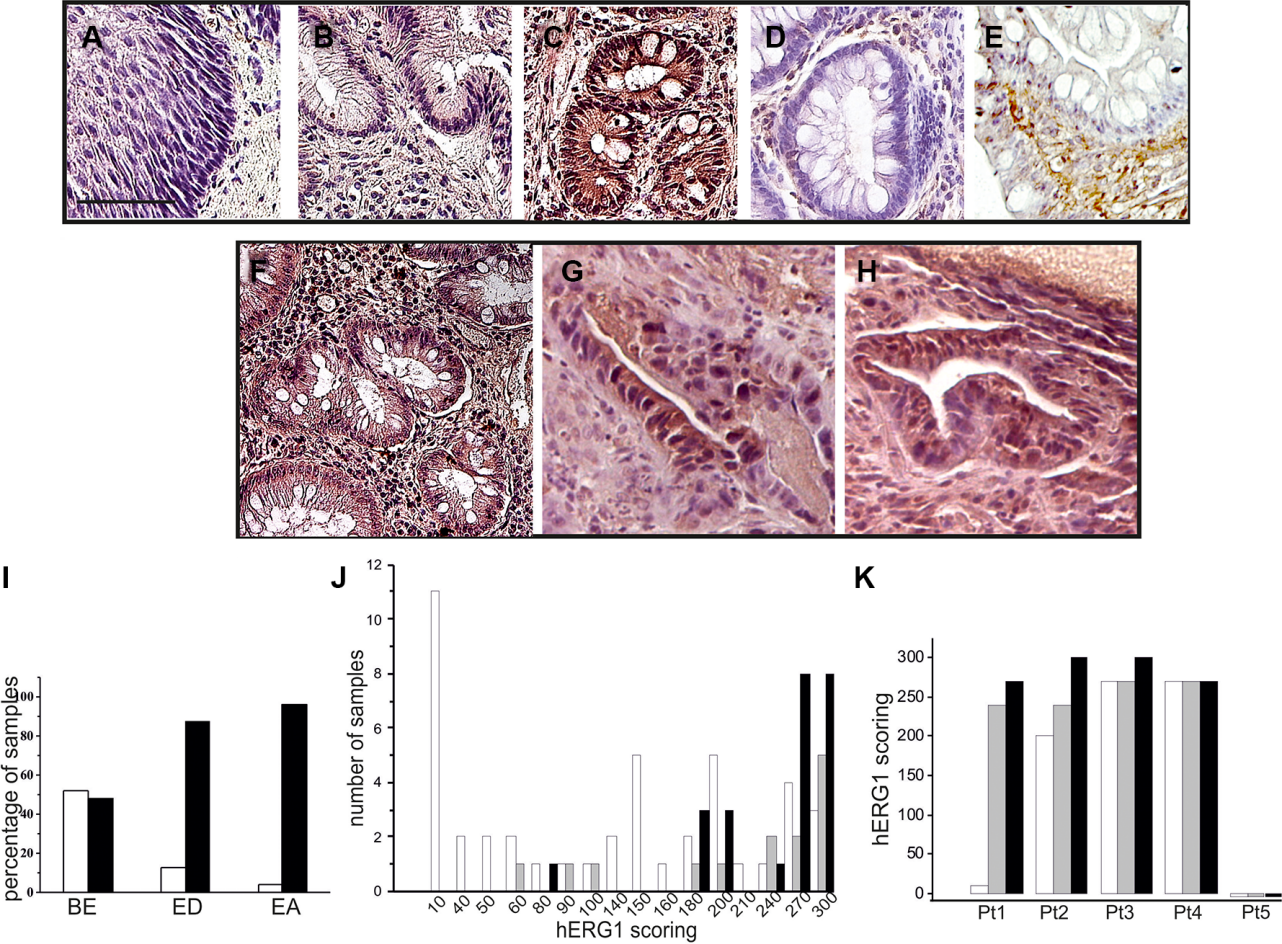
hERG1 expression in human esophageal carcinogenesis IHC was performed as described in Materials and Methods with anti-hERG1 monoclonal antibody. (**A**) Normal esophagus. (**B**) Esophagitis. (**C**) Representative BE sample expressing the hERG1 protein. (**D**) Representative BE sample negative for hERG1 expression. (**E**) Representative example of IHC performed with anti-hERG1B antibody (Dival Toscana Srl, Sesto Fiorentino) in a BE sample. (**F**–**H**) BE (panel F), Esophageal Dysplasia (ED, panel G) and Esophageal Adenocarcinoma (EA, panel H) of a representative patient whose lesion progressed towards malignancy. Original magnification 20×. Scale bar: 100 μm. (**I**) Histogram summarizing hERG1 expression in the three different groups of samples analyzed (BE, ED and EA). White bars: hERG1 negative samples, Black bars: hERG1-positive samples. (**J**) Histogram summarizing hERG1 scoring in the different groups (BE, ED and EA). Samples were scored as described in Materials and Methods. Analysis performed using 2-sided Student's *T* test revealed statistically significant differences between BE and ED (*p* = 0.023) and between BE and EA (*p* < 0.0001). (**K**) Histogram summarizing hERG1 scoring in the different samples (BE, ED and EA) of 5 representative patients. White bars: BE; Grey bars: ED; Black bars: EA.

In a previous study, performed in a relatively small cohort of patients, we showed that BE samples are positive for hERG1 expression, whereas both normal esophageal mucosa and GERD samples without or with esophagitis are hERG1 negative [16]. We first confirmed these data in a larger cohort of BE samples (125 patients instead of 27), applying a different antibody. Instead of the anti hERG1 polyclonal antibody, we used an anti-hERG1 monoclonal antibody (Mab-hERG1, Dival Toscana Srl; Sesto Fiorentino, Italy) which recognizes an extra-cellular epitope (the S5-P loop) of the hERG1 protein and can be used without permeabilization. Overall, the Mab-hERG1 gives a clearer immunohistochemical signal, with easiness of interpretation [17]. As shown in Figure 1, a clear positivity for hERG1 can be observed in the metaplastic cells characterizing BE lesions (Figure 1C), while no hERG1 expression could be detected either in normal squamous epithelium (Figure 1A) or in areas displaying signs of esophagitis (Figure 1B). Overall, hERG1 was expressed in 48% (60/125) of BE samples (see also the black bar addressed as “BE” in Figure 1I). A representative example of a hERG1 positive BE sample is reported in Figure 1C, while a hERG1 negative sample is in Figure 1D. Two main isoforms of the hERG1 protein (the full length hERG1A and the N-terminus deleted hERG1B) exists and are simultaneously expressed in some types of cancer [18]. Since the Mab-hERG1 does not discriminate between hERG1A and hERG1B, we performed an IHC assay using the anti-hERG1B antibody to determine whether such isoform is expressed in BE lesions. As evident in Figure 1E, no hERG1B expression can be detected in BE metaplastic cells, whereas a strong hERG1B signal is evident in muscle cells of the surrounding stroma, as expected [19, 20].

Overall, these results confirm that hERG1, and in particular the hERG1A isoform, is expressed in metaplastic cells characterizing BE lesions [16], and show the robustness (lower background and easiness of interpretation) of using the Mab-hERG1.

We also defined a scoring system, taking into account both the percentage of labeled cells and the staining intensity (from 0 to 3). The complete score was obtained by multiplying the two values and was therefore ranging from 0 (negative) to 300. Considering only positive samples (i.e. with scores >1), it emerged that BE samples have a median score equal to 145.0 ± 86.0 (*n* = 60). Sixteen ED and twenty-five EA cases were also collected and analyzed. Five cases, for which the entire progression from BE to ED and then to EA was available, were fully analyzed and scored. Pictures relative to one of such cases are shown in Figure 1F–1H. Overall, hERG1 was expressed in 87.5% (14/16) ED and 96% (24/25) EA (Figure 1I). Applying the scoring system described above, BE samples showed a significantly lower median score (145.0 ± 86.0), compared to ED (255.0 ± 70.7, BE *vs* ED *p* = 0.023) and EA (270.0 ± 48.4, BE *vs* EA *p* < 0.0001) (Figure 1J). We analyzed the BE, ED and EA scorings of those five cases for which the entire pathology progression was available: hERG1 positive BE samples showed a trend to increase hERG1 expression during progression (Figure 1K and Supplementary Table S1).

Collectively, these data show that hERG1 is expressed in roughly half of BE samples, and its expression quantitatively increases during BE progression to adenocarcinoma.

### hERG1 is expressed in BE lesions of BE mouse models

**Figure 2:**
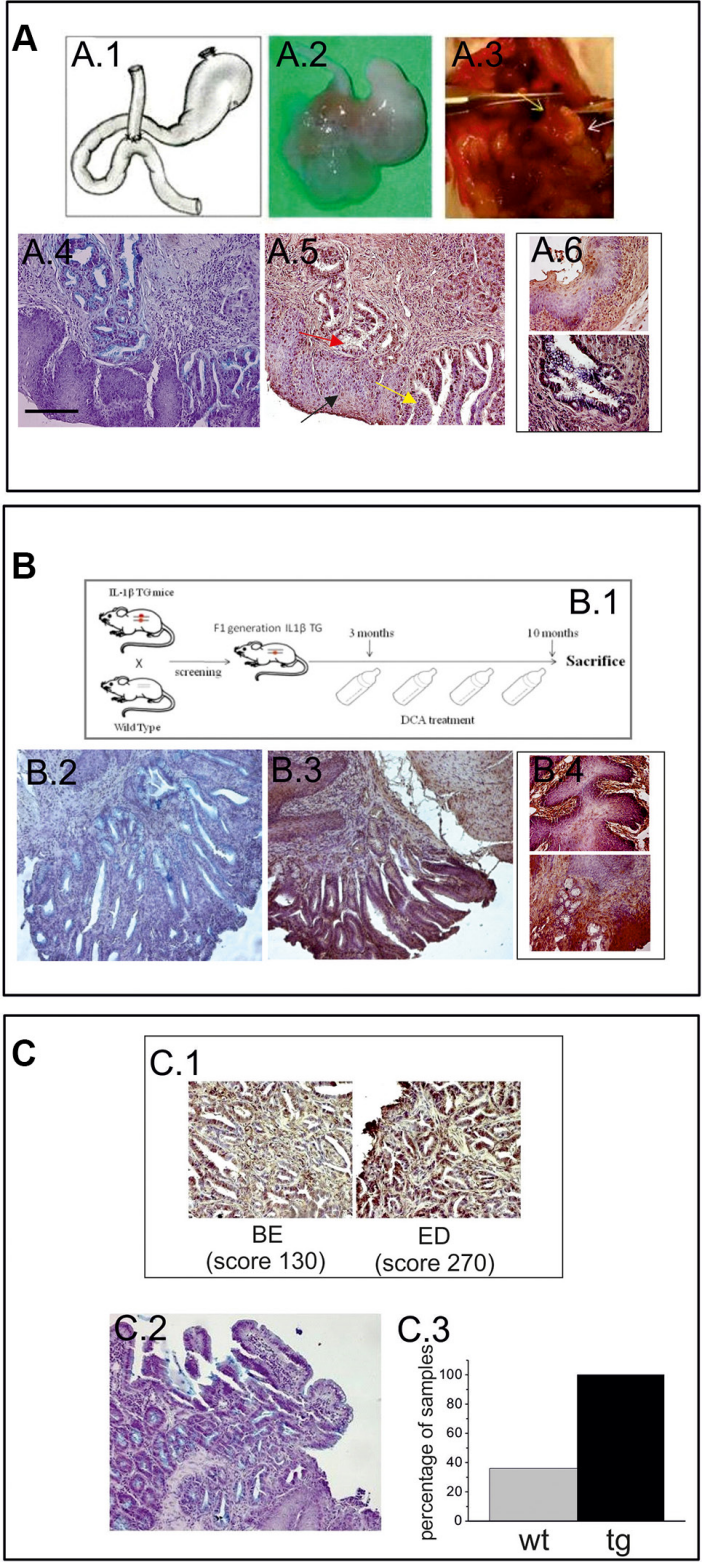
hERG1 is up-regulated in BE mouse models: (A.1) Surgical procedure: drawing of the EJA performed in this study (**A.2**) Representative specimen from an operated animal, showing EJA. (**A.3**) EJA at the moment of the sacrifice: the yellow arrow indicates the esophagus, the white one indicates the atrophic stomach. (**A.4**) Alcian Blue staining performed on Balb-C mice in order to detect goblet cells. (**A.5**) IHC performed on Balb-C mice, in order to evaluate hERG1 expression, indicates that hERG1 is not expressed in normal esophageal tissue (black arrow) while it is up-regulated in metaplasia (red arrow) and dysplasia (yellow arrow). (**A.6**) Higher magnification microphotographs showing IHC performed in normal squamous tissue (upper panel) and BE (lower panel). (**B.1**) Birth and screening of IL-1β TG mice F1 generations: each founder mouse is bred with a Wild Type partner. The IL-β TG mice are treated with 0,2% DCA for 7 months and then sacrificed. Alcian Blue (**B.2**) and IHC (**B.3**) staining performed on IL-1β TG mice DCA-treated in order to detect BE lesions and to evaluate hERG1 expression. (**B.4**) Higher magnification microphotographs showing IHC performed in normal squamous tissue (upper panel) and BE (lower panel). (**C.1**) A detail of metaplastic area (left) and dysplastic area (right) of a Balb-C mouse sample. hERG1 is up-regulated in BE lesions (with low score) and in dysplasia (with high score) as detailed in Results. (**C.3**) Alcian Blue staining shows BE lesion in hERG1 TG mice Scale bar 100 μm. Original magnifications, 20× (A.4, A.5, B.2, B.3, C.1, **C.2**)) and 40× (A.6, B. 4). C.3) Histogram showing the percentage of mice that developed BE. Grey bar: wild type operated mice (wt); black bar: hERG1 TG mice operated as described in Materials and Methods. IHC performed on hERG1 TG mice confirm the HERG1-positive labelling.

We also developed, and tested for hERG1 expression, different BE mouse models, in which BE lesions were induced either surgically or chemically. For the “surgical” model, mice (either CD1 or Balb-C) were subjected to an esophago-jejunal anastomosis (EJA) (Figure 2A.1–2A.3). Mice were sacrificed 9 to 12 months after surgery and the esophagus, the stomach and intestine were removed, and analyzed for the detection of BE lesions through hematoxylin and eosin as well as Alcian blue staining (Figure 2A.4). All the operated animals showed signs of gastric mucosal atrophy due to the EJA (Figure 2A.2 and 2A.3). 4 out of 11 (36%) operated animals developed histologically detectable intestinal metaplasia in the lower esophagus, indicative of BE (Figure 2A.5, red arrow). One mouse also showed signs of ED in an area surrounding a BE lesion (yellow arrow in Figure 2A.5).

The “chemical” model was based on chronic esophageal inflammation: IL-1β transgenic (TG) mice received, in their drinking water, 0.2% deoxycholic acid (DCA) for 7 months of treatment (see the scheme in Figure 2B.1). Four out of 10 (40%) IL-1β TG DCA-treated mice developed histologically detectable BE lesions (Figure 2B.2).

An IHC analysis was performed in order to evaluate the expression of hERG1 in BE lesions of both mouse models. hERG1 was absent in either normal (see black arrow in Figure 2A.5, Figure 2A.6 upper panel, Figure 2B.3 and Figure 2B.4 upper panel) or inflamed (esophagitis) esophageal tissue in both models (Supplementary Figure S2), while it is up-regulated in BE lesions arising in both types of models (Figure 2A.5, Figure 2B.3, Figure 2B.4 lower panel and Figure 2C.1 left panel) (mean score = 130.0 ± 28.7 in the surgical model and mean score = 113.0 ± 37 in the chemical model) as well as in the dysplasia observed in the surgical model (mean score = 270.0) (Figure 2C.1 right panel).

Finally, we tested the EJA surgical procedure in mice overexpressing hERG1. To this purpose, we operated FVB hERG1 transgenic (TG) mice whose hERG1 over-expression in the GI tract was proven in [21]. To detect intestinal metaplasia, the Alcian Blue staining was applied (Figure 2C.2), as above. All (4/4) the FVB hERG1 TG mice developed BE lesions 9–12 months after surgery (Figure 2C.3).

### hERG1 is an indicator of BE progression to adenocarcinoma

**Figure 3:**
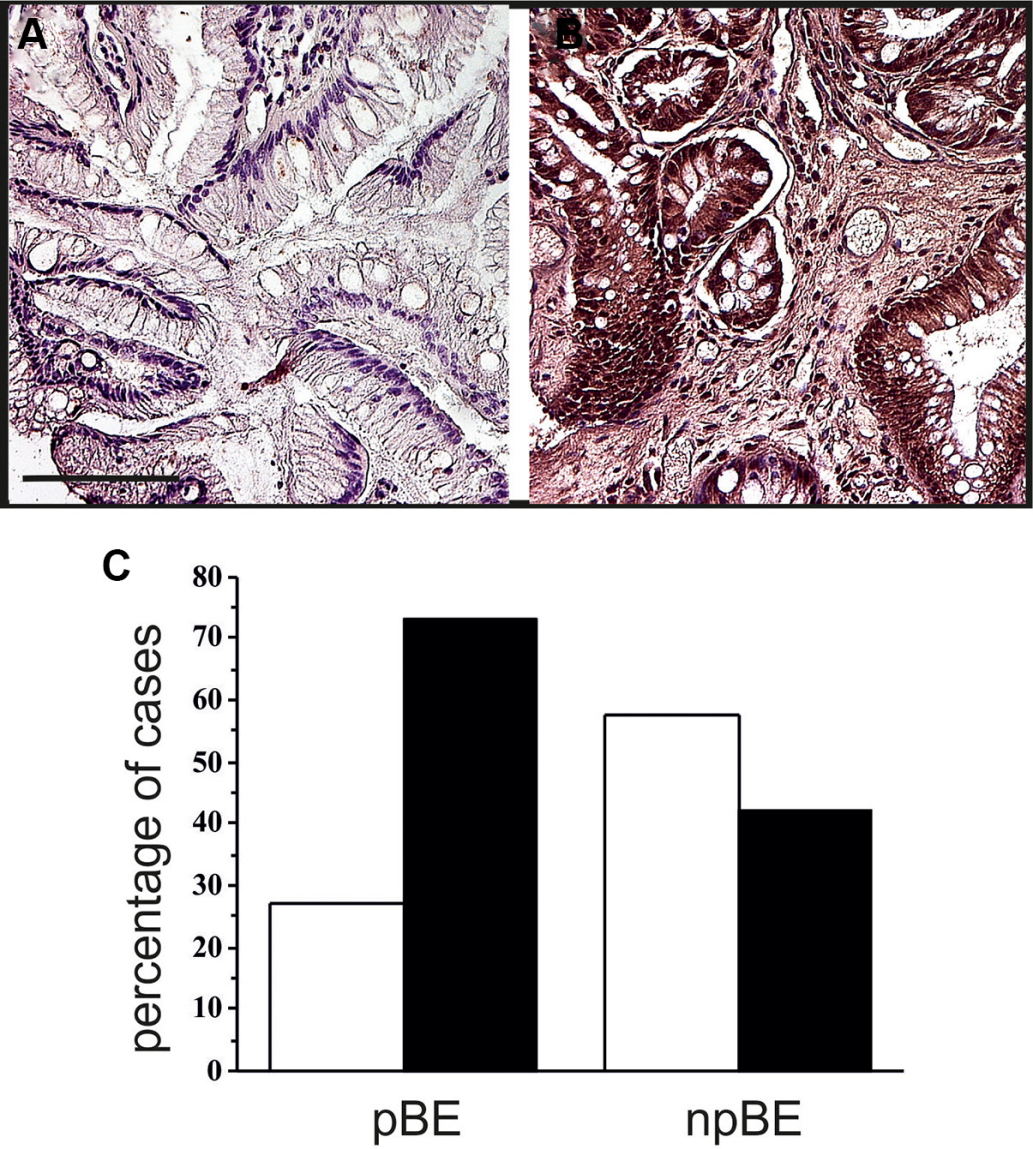
hERG1 expression along tumor progression (**A**) Non-progressed BE. The representative sample here reported is negative for hERG1 expression. (**B**) Progressed BE. A representative hERG1-positive sample is shown. Original magnification 20×. Scale bar: 100 mm. (**C**) Histogram showing hERG1 expression in progressed (pBE) and non-progressed BE (npBE). White bars: hERG1 negative; Black bars: hERG1 positive.

We then tested whether hERG1 might represent a biomarker of tumor progression in BE. A case-control study design was chosen to analyze the potential association between a rare medical condition (EA in BE) and a relatively common marker (hERG1 expression). We analyzed 104 samples with a bioptic diagnosis of BE at the onset and a follow-up of at least 10 years, provided from different institutions in Italy. Both samples that progressed towards ED and/or EA in the follow-up time (pBE, *n* = 26) and samples belonging to patients whose lesions did not progress to EA (npBE, *n* = 78) were collected. For this analysis samples were simply scored as “negative” or “positive” for hERG1 expression. Representative examples of a npBE sample, which does not express the hERG1 protein, and of a pBE sample, which is positive for hERG1 expression are shown in Figure 3A and 3B, respectively. Overall, hERG1 is expressed in 19 out of 26 pBE (73.1%) while only 42.3% (33 out of 78) npBE expressed hERG1 (Figure 3C). The analysis of the case-control study indicated a statistically significant association between BE hERG1 expression status and risk of progression to adenocarcinoma (odds ratio = 3.70, 95% CI: 1.40–9.82; *P* = 0.006) (Table 1). It is worth noting that hERG1 positivity in pBE is not comparable with that observed in the whole series, since, in the case-control study, we only focused on selected patients whose lesions progressed towards ED or EA. The distribution analysis of the samples according to a more complex scoring system did not highlight any trend (see Supplementary Figure S1).

**Table 1:** Statistical analysis for the case-control retrospective study

	npBE (%)	pBE (%)	OR	95% CI	*P* value (Likelihood ratio test)
**hERG1−**	45 (57.7%)	7 (26.9%)	3.70	1.40–9.82	*P* = 0.006*
**hERG1 +**	33 (42.3%)	19 (73.1%)			

Abbreviations: npBE: not progressed BE, pBE: progressed BE.

We also analyzed hERG1 expression in either ED or EA lesions (which available) of the 26 pBE patients. Those 20 BE samples that were hERG1 positive maintained their positivity and increased the scoring in the ED/EA lesions (mean values 161.8 ± 31.3 and 237.3 ± 16.9, respectively). The ED/EA lesions of four hERG1 negative BE cases became positive (Table 2). This made the percentage of positive cases to increase from 76.9% to 92.3%. The significance of this result was confirmed by the McNemar's Test (*p* = 0.045, Supplementary Table S2).

**Table 2:** hERG1 expression in patient-matched pBE, ED/EA included in the case-control study

	pBE	ED	EA
**BS1**	+	N/A	+
**BS2**	+	N/A	+
**BS3**	+	+	+
**BS4**	+	N/A	+
**BS5**	+	+	N/A
**BS6**	−	+	N/A
**BS7**	+	N/A	+
**BS8**	+	+	+
**BS9**	−	N/A	+
**BS10**	+	+	+
**FI1**	+	N/A	+
**FI2**	+	N/A	+
**FI3**	+	N/A	+
**FI4**	+	N/A	+
**FI5**	+	N/A	+
**FI6**	+	N/A	+
**SI1**	+	+	N/A
**SI2**	+	+	N/A
**SI3**	−	−	N/A
**SI4**	−	−	−
**SI5**	+	+	+
**SI6**	+	+	N/A
**SI7**	−	+	N/A
**SI8**	+	+	N/A
**SI9**	+	+	N/A
**SI10**	+	+	N/A

Abbreviations: N/A: histological sample not available for IHC evaluation.

### scFv-hERG1-alexa488 test on fresh surgical esophageal biopsies

**Figure 4:**
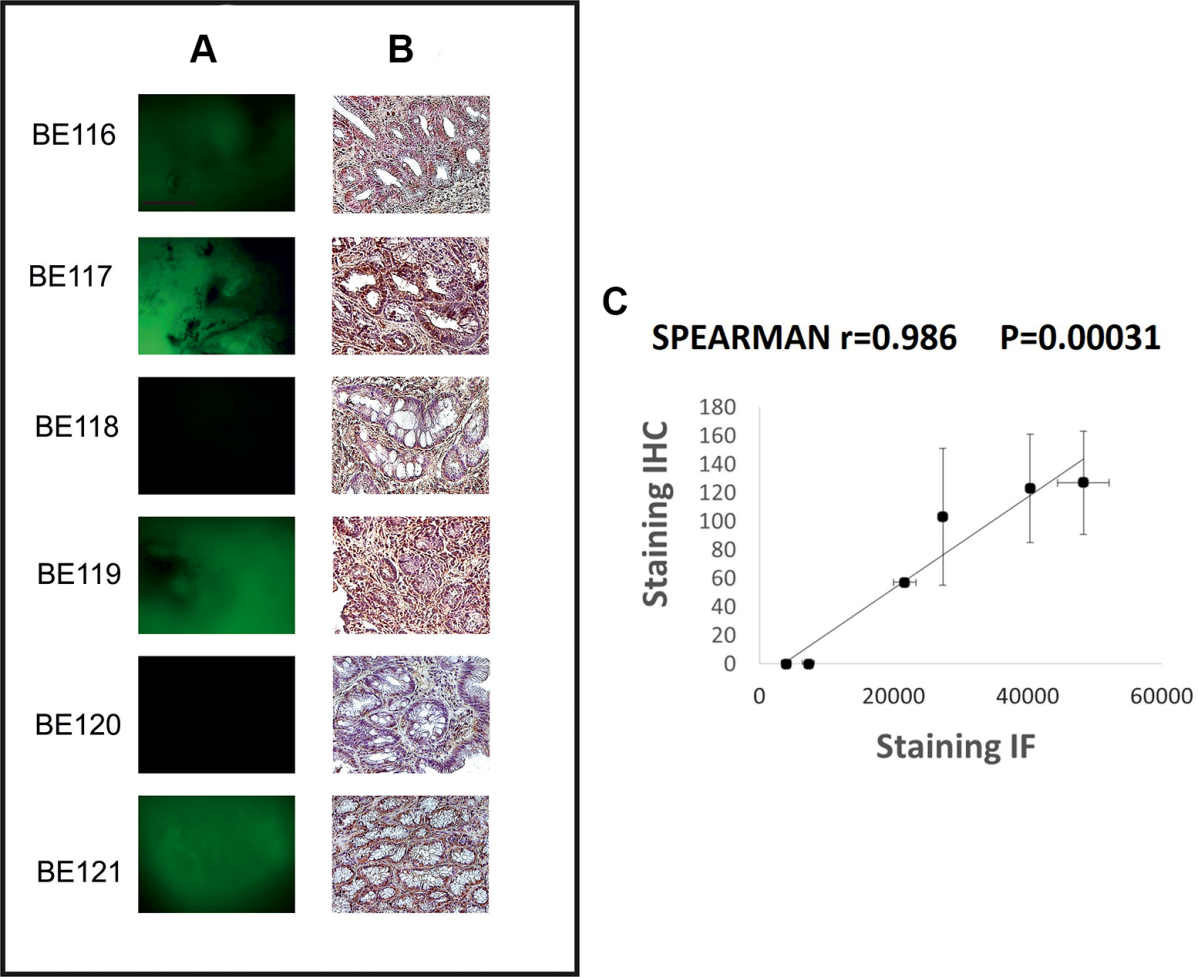
scFv-hERG1-Alexa488 test on fresh surgical esophageal biopsies Representative images of Immunofluorescence with scFv-hERG1-Alexa488 (**A**) and corresponding immunohistochemical analysis with anti-hERG1 monoclonal antibody (**B**). Scale bar: 100mm. Original Magnification: 10× (A), 20× (B). (**C**) Scatter plot, with standard error, showing the concordance between IHC and IF. Each point represents a BE patient. IHC staining: hERG1 expression was evaluated applying, for three areas of BE lesion from each sample, an immunohistochemical score obtained through the combination of the estimate of the percentage of immunoreactive cells (quantity score) with the estimate of staining intensity (staining intensity score). The raw data were converted to the combined score by multiplying the percentage and staining intensity values, obtaining a value between 0 and 300 for each area and finally the mean of the three areas was computed for each sample (mean score: 57, 103, 0, 127, 0, 123); IF staining: for each sample the measures of three different areas of the image with the strongest fluorescent scFv-hERG1-Alexa488 was performed with Image J. The mean of the three measures, obtained from negative control, was calculated and this value was subtracted in each result of the three different areas of other samples and then for all samples the average was computed (obtained values: 21.696, 27.361, 7.320, 48.346, 4.041, 40.367).

The data described above indicated that hERG1 expression could help in identifying patients at higher risk of progressing from BE to adenocarcinoma. We therefore tested the immunoreactivity of a scFv-hERG1, derived from the anti-hERG1 monoclonal antibody (Duranti, Sette, manuscript in preparation), labeled with the Alexa488 fluorophore. With the aim of further testing the scFv-hERG1-Alexa488 in endoscopy to discriminate between hERG1-positive and hERG1-negative BE patients, we tested such antibody fragment on fresh endoscopic mucosal samples from BE patients. IHC with the Mab-hERG1 was carried out on parallel paraffin-embedded samples. Figure 4 shows representative images of either fresh samples (panel A) or fixed samples (panel B). Samples with an intense IHC signal, also displayed a positive IF signal. The mean IHC and IF values, calculated as described in the figure legend, were used to perform a statistical analysis, applying the Spearman Correlation Test. A statistically significant value was obtained (*P* = 0.00031), with a coefficient of 0.986. Figure 4C shows a scatter plot with the concordance between IHC and IF staining.

## DISCUSSION

EA has a very poor prognosis mainly due to lack of early diagnosis and complex therapeutic approaches. BE is the only well-known precursor of EA, and for this reason endoscopic surveillance protocols have been proposed for BE patients. However, due to the wide variation in the estimates of the progression rate from BE to EA (0.07%–3.6%), and the lack of validated biomarkers of BE progression, a higher number of BE patients than necessary are subjected to invasive endoscopic and bioptic procedures. This justifies the fact that currently there are no evidences that BE screening effectively reduces EA incidence and mortality [22].

In search of novel BE biomarkers, we here provide evidence that hERG1 channels can be considered novel markers of progression in BE patients. In particular, we applied a specific anti-hERG1 monoclonal antibody, which recognizes an extracellular epitope of the protein and can hence be used in IHC without permeabilization. Through this procedure, we showed that (a) the hERG1 protein is overexpressed in BE, confirming a previous pilot study [16], and that the prevalent hERG1 isoform is hERG1A, as occurs in the majority of solid cancers examined so far [15, 23, 24]. On the other hand, we found a strong expression of the hERG1B isoform in smooth muscle cells of the submucosa, as described in rodents [19, 20, 25]. After applying an IHC scoring system, based on the contemporary assessment of the percentage of labelled cells and the signal intensity, we also proved that (b) hERG1 expression increases along BE progression to ED and EA. This was proven in separate cohorts of BE, ED and EA samples (Figure 1I and 1J) as well as in a subset of patients whose BE lesions progressed to ED or EA and for which matched BE and ED/EA samples were available (Figure 1K, Table 2 and Supplementary Table S1).

We then tested whether hERG1 expression could be detected in different BE mouse models, to be exploited in the future for preclinical *in vivo* imaging and pharmacological studies. In particular, we confirmed hERG1 expression in the metaplastic cells arising in BE lesions of two different mouse models: a surgically-induced and a chemically-induced model. Moreover, in one mouse in which BE progressed to ED, the scoring of hERG1 expression increased. Interestingly, we also found that the percentage of mice developing BE after the surgical procedure greatly increased (from 36% to 100%) in transgenic mice which over express the *hERG1* gene in the GI tract, including the esophagus [21]. Although these results were obtained in a small subset of animals, they might suggest a potential causative role of hERG1 in BE pathogenesis, a topic to be further studied in the future.

The main translational result of the present paper was that obtained through a case-control study, in which the association between hERG1 expression and development of adenocarcinoma was evaluated in samples from patients’ biopsies collected at the diagnosis of BE. Patients enrolled in the study had a follow up of at least 10 years, so that the progression to EA had been adequately monitored. Our data demonstrated a statistically significant association between hERG1 expression status in BE patients and risk of progression to EA (odds ratio = 3.70, 95% CI: 1.40–9.82; *P* = 0.006) (Table 1). In other words, hERG1 represents a progression factor that contributes to identify high-risk BE patients. Nevertheless, since the percentage of hERG1-positive pBE samples is not strikingly different from hERG1-positive npBE (although the statistically significant results reached), extreme caution should be applied, to take into account false-positive samples.

Over the years it has become clear that hERG1 plays a relevant role in several types of cancer. In particular, hERG1 is a marker of advanced stage in colorectal cancers [23], where it contributes to identify high risk TNM stage II patients [17, Muratori L, Petroni G et al., submitted]. In gastric [15] and pancreatic cancers [26] hERG1 is a prognostic biomarker also in early stages cancers, contributing to identifying patients with worse prognosis. Such different scenarios should be related to the modulatory effects that hERG1 exerts on intracellular signaling pathways which control cell proliferation, survival, invasiveness, VEGF-A secretion and in turn angiogenesis (reviewed in [25]). The mis-expression of hERG1 in esophageal mucosa, even at early stages of esophageal cancerogenesis, could in turn modify cellular behavior switching on survival and pro-angiogenic signals, which in turn promote proliferation of BE metaplastic cells [27].

In the field of esophageal diseases, hERG1 could be used to better identify BE patients at high risk to progress alone or in combination with other progression indicators. Nowadays, the detection of dysplasia is the commonest histopathological progression marker. Besides being a late marker of progression, it is also affected by several biases such as biopsy sampling error, subjective evaluation and possibility of regression, at least in low grade ED. For these reasons, other progression markers have been proposed such as 9p and 17p loss of heterozigosity (LOH) and aneuploidy [5–10, 28]. Recently, P53 immunohistochemical evaluation has been proposed as a progression marker, although the wide variation in the expression as well as the high false-positive and false-negative rates limit its usefulness (reviewed in [29]). The immunohistochemical evaluation of hERG1 on biopsies obtained during endoscopic procedures would help to better diagnose BE patients at high risk of progressing towards EA. The possibility of including other BE progression predictive biomarkers in the IHC procedure could be of great advantage, reducing false positive results. Overall, once clinically validated, a panel of biomarkers including hERG1 could help to design the most useful surveillance or treatment protocol.

Finally, to address the problems dealing with the clinical management of BE surveillance, we provided data which allow us to propose a different and simpler approach. We tested an Alexa-conjugated scFv-hERG1 antibody which recognizes an epitope located extracellularly and can hence be used without permeabilization, on freshly collected, live endoscopic BE biopsies. The good concordance between IF (with the scFv-hERG1-Alexa488) and IHC (with the Mab-hERG1) allows us to propose the scFv-hERG1-Alexa488 as a novel tool for optical *in vivo* imaging. For *in vivo* imaging procedures, scFvs are preferred with respect to whole antibody molecules, due to their small size (25–30 kDa), that allows them to penetrate solid tumor mass. Indeed, Sturm et al. [30] recently showed that a fluorescently-labelled peptide can be safely administered to patients and revealed by confocal endomicroscopy to successfully identify ED and EA. Moreover, scFvs are ideal vectors for the delivery of agents such as radionuclides, enzymes, drugs or toxins *in vivo* [31–34]. After proper testing for safety, the scFv-hERG1-Alexa488 might be used in the future within standard endoscopic procedures and novel targeted-imaging techniques (reviewed in [13]).

Overall, data reported in the present paper indicate that hERG1 identifies patients at higher risk to progress towards EA, and open the possibility of developing a new protocol for the surveillance of BE patients by the *in vivo* direct detection of hERG1-positive and hERG1-negative BE patients, using the scFv-hERG1-Alexa488.

## MATERIALS AND METHODS

### Patients and tissue specimens

Tissue samples were obtained from different institutions belonging to GIRCG (Department of Clinical and Experimental Medicine, University of Florence; Pathology Division, Azienda Ospedaliero-Universitaria Senese; Pathology Division, Borgo Trento Hospital, Verona; Pathology Division, Morgagni-Pierantoni Hospital, Forlì; Pathology Division, Esine Hospital, ASL Vallecamonica Sebino; Insitute of Pathology, Spedali Civili, Brescia). A total of 125 BE, 16 ED and 25 EA paraffin-embedded samples were collected.

Diagnosis and histological grading were assessed in all cases using standard criteria by experienced pathologists (LM, CV, AT, LS, MC, MS and VV).

### Immunohistochemistry

Immunohistochemistry was performed as previously reported [17] using an anti-hERG1 monoclonal antibody directed against the S5-pore region (Dival Toscana Srl, Sesto Fiorentino, Italy) at 1:200 dilution. Immunohistochemistry for hERG1B was performed following the same protocol with the exception of permeabilization, carried out by adding Triton X-100 to the blocking solution. The antibody (purified polyclonal anti-hERG1B antibody, Dival Toscana Srl, Sesto Fiorentino, Italy) was used at a final dilution 1:1000. Slides were incubated overnight at 4°C and immunostaining was performed with a commercially available kit (PicTure Max kit and DAB, Invitrogen; Carlsbad CA, USA).

### Scoring assessment

Samples were evaluated applying a scoring system frequently used for cytoplasmic and membrane proteins [26]. Such scoring system combines the estimate of the percentage of positive cells with the staining intensity. Staining intensity was rated on a scale of 0–3, with 0 = negative; 1 = weak; 2 = moderate, and 3 = strong. The raw data were converted to the combined score by multiplying the percentage and staining intensity values, obtaining a value between 0 and 300 for each sample. Only samples with a complete score equal to 0 were considered negative. All the samples were evaluated using Leica DMR light microscope (Leica; Wetzlar, Germany). Samples were evaluated and scored by three independent operators (EL, TL and JI).

### Case-control study

Cases were defined as BE subjects whose lesions progressed towards dysplasia/adenocarcinoma while Controls were BE patients, with at least a followup visit completed during the last 10 years, whose lesions didn’t progress at the last time when they were examined. Since there may be a considerable lag time between the diagnosis of BE and the progression towards EA, the date of BE diagnosis was defined as index date. Three controls were individually matched per case for age at the index date and gender. Controls were randomly chosen, without replacement, between all individuals with a followup duration equal to or longer than the interval between index date and EA diagnosis in the corresponding case.

We calculated that, for a twosided alpha error equal to 5%, with 15 cases available, a casecontrol ratio equal to 1:3, and a prevalence of hERG1 expression between controls equal to 10%, the study will have a power of 80% against a minimal detectable relative risk (odds ratio) of 6.7. According to our previous data, such a strong association should be plausible.

### scFv-hERG1-Alexa488 test on fresh surgical esophageal biopsies

Endoscopic mucosal samples from six BE patients were collected from the Department of Surgery and Translational Medicine, University of Florence, under a protocol approved by the Local Ethic Committee. Two consecutive samples of fresh biopsies of about 2–3 mm in diameter were harvested during endoscopic procedures from the site of BE lesion of each patient, checking each site of biopsy with enhanced endoscopy (NBI – Olympus). Immediately a sample was immersed in PBS 1X and used for experiments with scFv-hERG1-Alexa488 (see below); the other sample was put in 3.7% formaldehyde for fixation, paraffin embedding and immunohistochemical staining with anti-hERG1 monoclonal antibody. Immunohistochemistry was performed as previously described [17] without permeabilization and hERG1 expression was evaluated applying, for three areas of BE lesion from each sample, an immunohistochemical score obtained through the combination of the estimate of the percentage of immunoreactive cells (quantity score) with the estimate of staining intensity (staining intensity score). Staining intensity was rated on a scale of 0–3, with 0 = negative; 1 = weak; 2 = moderate, and 3 = strong. The raw data were converted to the combined score by multiplying the percentage and staining intensity values, obtaining a value between 0 and 300 for each area and finally the mean of the three areas was computed for each sample.

Experiments with scFv-hERG1-Alexa488 were performed using a recombinant scFv-hERG1 (patent N° FI2014A000189) directed against the S5-pore region and derived from the anti-hERG1 monoclonal antibody (Duranti, Sette et al, manuscript in preparation). Briefly, the variable immunoglobulin domains of heavy and light chain of hERG1-mAb were cloned into the expression vector pPIC9K to produce a His-tag protein in *Pichia Pastoris*. For further applications, scFv-hERG1 antibody large-scale production was necessary: for these purposes, large-scale cultures were set up following and validating a protocol useful both for batch and bioreactor protein production. The scFV was labeled with Alexa Fluor 488 Microscale Protein Labeling kit (Thermofisher Scientific; CA, USA). For staining live bioptic samples the following protocol was applied: fresh, non-fixed tissue samples, orientated with the mucosal layer facing upwards, were laid on an agarose liquid surface homogeneously distributed on Cell Culture Chamber Slide (Euroclone; Milan, Italy). Biopsies were incubated in the dark with scFv-hERG1-Alexa488, diluted 1:20 in PBS 1×, at 4°C overnight. The following day samples were washed three times with PBS 1X before and after performing Hoechst staining (1:1000 in PBS 1×) for 30 min. The negative control was considered one of the sample before the incubation with scFv-hERG1-Alexa488.

In this study a directed light fluorescence microscope Leica DMR, (Leica; Wetzlar, Germany) was used. During the scanning, after the focus of the sample performed through Hoechst stain, the laser delivers the 488 nm excitation wavelength to measure the scFv-hERG1-Alexa488 signal. The images were captured at the same time of exposure for all biopsies for later download and processing. The images were analyzed using Image J software. The image with the strongest fluorescent scFv-hERG1-Alexa488 signal was chosen for each sample and the measure of three different areas was performed. The mean of the three measures, obtained from negative control, was calculated and this value was subtracted in each result of the three different areas of other samples and then for all samples the average was computed.

### Induction of BE in mice

*In vivo* experiments were performed at the Laboratory of Genetic Engineering for the Production of Animal Models (LIGeMA) at the Animal House of the University of Florence (Ce.S.A.L.). All experiments have been carried out in accordance with the Principles of Laboratory Animal Care (directive 86/609/EEC).

Balb-C, CD-1 mice (2–4 months of age, weighing 24–29g) and FVB hERG1 transgenic mice (2–4 months of age, weighing 23–30g), overexpressing hERG1 in GI tract [18], were operated to perform gastro-jejunal anastomosis (EJA), inducing gastro duodenal mixed reflux. Anesthesia was performed by an intraperitoneal injection of Avertin 2.5% (16 μg/g body weight).

Surgery was performed by placing the mouse on a small surgical table and, after a 2–3 cm median incision of the skin and peritoneum, the esophagogastric junction was exposed. Distal esophagus was sectioned after clamping in order to prevent esophageal retraction and the gastric side ligated with dexon 6/0 suture and the continuity of the gastrointestinal tract restored by end-to-side esophago-jejunal anastomosis with 7/0 silk suture (Figure 2A.1– 2A.3). After repositioning of the viscera the abdominal wall was closed with nylon 4/0. Mice were sacrificed after 9 and 12 months. Furthermore, another set of experiment was performed using a chemical model of BE treating four 3-month-old 10 IL-1β TG mice [35] with 0.2% deoxycholic acid (DCA) in the drinking water (pH 7.0). Human IL-1β transgenic mice were generated by targeting expression of hIL-1β to the esophagus using the Epstein Barr virus promoter, as proven in [35], and screened using human IL-1β specific PCR primers. 7–8 months after the beginning of the DCA-treatment mice were sacrificed.

After the animal sacrifice the stomach and esophagus were removed and fixed in 4% formalin for 24 hours. Thereafter, samples were processed for paraffin embedding and 7 μm longitudinal cut sections were obtained through a microtome and put on positive-charged slides.

Samples were stained with Hematoxylin/Eosin and Alcian Blue standardized protocols to detect goblet cells and then observed under a light microscope. Moreover, samples were stained with anti-hERG1 polyclonal antibody, in order to evaluate the expression of hERG1 channel. The antibody was diluted in UltraVBlock (Bio-Optica; Milan, Italy) in PBS1:10 (v/v) at a final dilution 1:200 [23].

### Statistical analysis

For the case-control study, the estimates of the odds ratio and its 95% confidence interval were obtained from a logistic regression model. Statistical analyses were performed by LB using SAS version 9.2 (SAS Institute, Cary, NC).

The comparison between hERG1 expression in paired pBE and ED/EA was evaluated by McNemar's test (*P* < 0.05).

The association between the value of scFv-hERG1-Alexa488 signal and immunohistochemical score of anti-hERG1 monoclonal antibody was analyzed using the Spearman correlation test. *P* < 0.05 was considered statistically significant.

## SUPPLEMENTARY MATERIALS FIGURES AND TABLES


