# Supplementary material for: hERG1 behaves as biomarker of progression to adenocarcinoma in Barrett's esophagus and can be exploited for a novel endoscopic surveillance
Source: Oncotarget. 2016 Aug 9;7(37):59535–47. doi: 10.18632/oncotarget.11149 (PMC5312329; doi:10.18632/oncotarget.11149)
Supplement: Supplementary file 1 [file oncotarget-07-59535-s001.pdf]

hERG1 behaves as biomarker of progression to adenocarcinoma in Barrett's esophagus and can be exploited for a novel endoscopic surveillance

Supplementary Materials

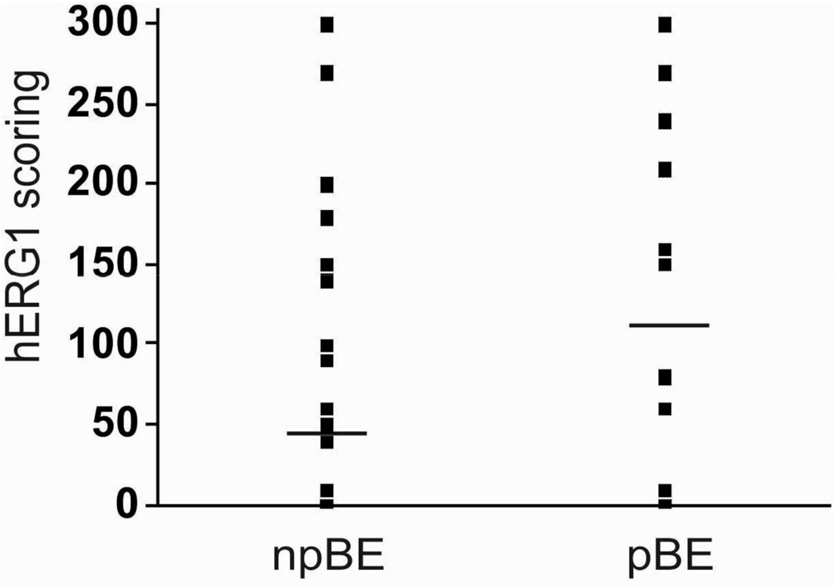

Supplementary Figure S1: Scatter Plot summarizing hERG1 scoring in the two different groups (npBE, pBE). Samples were scored as described in Materials and Methods. Each point represents a patient. Horizontal lines represent the mean value for hERG1 scoring in each group.

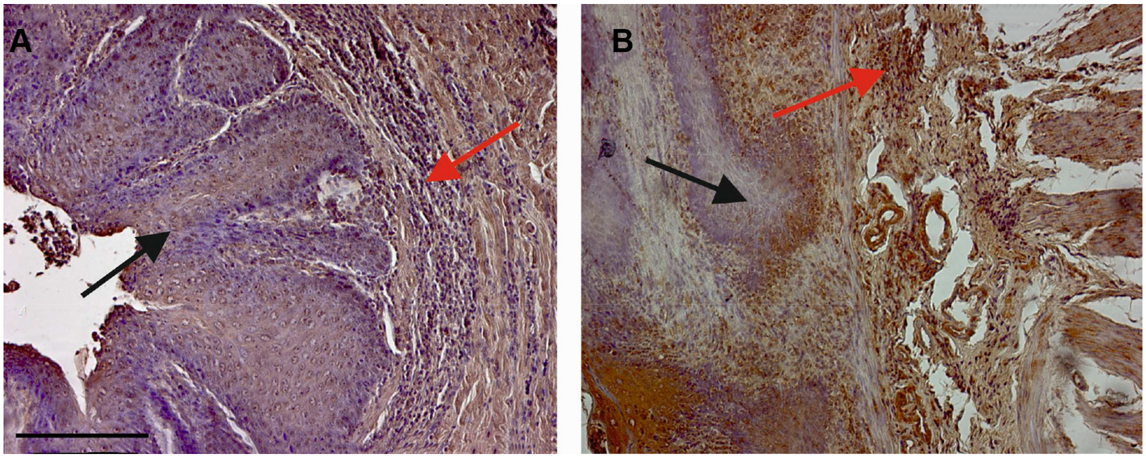

Supplementary Figure S2: Representative pictures of immunohistochemistry performed with anti-hERG1 polyclonal antibody (as described in Materials and Methods) showing that hERG1 protein is not expressed in normal esophageal tissue and in esophagitis. (**A**) "surgical model"; (**B**) "chemical model". Original magnification 20×. Scale bar: 100 µm.

Supplementary Table S1: hERG1 scoring in 5 patient-matched pBE, ED and EA

	pBE			ED			EA		
	**%**	**intensity**	**score**	**%**	**intensity**	**score**	**%**	**intensity**	**score**
**Pt1**	10	1	10	80	3	240	90	3	270
**Pt2**	100	2	200	80	3	240	100	3	300
**Pt3**	90	3	270	90	3	270	100	3	300
**Pt4**	90	3	270	90	3	270	90	3	270
**Pt5**	0	0	0	0	0	0	0	0	0

Supplementary Table S2: Results of hERG1 expression on 26 pBE and ED/EA matched samples from patients included in the case-control study

		pBE		
		**hERG1 –**	**hERG1 +**	**TOTAL**
**ED/EA**	**hERG1 –**	2	0	2
	**hERG1 +**	4	20	24
	**TOTAL**	6	20	26

hERG1 positivity: pBE = 76.9%, ED/EA = 92.3%; McNemar's test, $P = 0.045$.
